# Changes in walking speed following resistance training in people with multiple sclerosis: A systematic review and meta‐analysis

**DOI:** 10.1002/pmrj.13255

**Published:** 2024-09-23

**Authors:** Connor McManaman, Brianna Novak, Lorna Paul, Scott Rooney

**Affiliations:** ^1^ School of Health and Life Sciences Glasgow Caledonian University Glasgow UK

## Abstract

**Background:**

Reduced walking ability, especially decreased gait speed, is one of the most common and disabling impairments reported by people with multiple sclerosis (MS). Considering the impact of muscle strength on walking ability, resistance training may have the potential to improve walking speed in MS. Therefore, this systematic review and meta‐analysis aims to evaluate the effect of lower limb resistance training on walking speed in people with MS.

**Methods:**

Seven databases (CINAHL, MEDLINE, The Allied and Complimentary Medicine Database, Web of Science, Physiotherapy Evidence Database [PEDro], PsycINFO, and Sports Medicine and Education Index) were searched in March 2024 for studies that met the following eligibility criteria: randomized controlled trials investigating the effects of resistance training interventions on objective measures of walking speed in people with MS. Risk of bias was assessed using the PEDro scale. Meta‐analysis was performed to quantify intervention effect using a random effects model.

**Results:**

Twelve randomized controlled trials were included, reporting data on 425 individuals with MS. Participants had mostly relapsing–remitting MS (85%) and a mild–moderate level of disability (Expanded Disability Status Score 1.0–6.0). Results of the meta‐analysis (based on 7 of the included studies) indicated a significant yet variable improvement in walking speed in favor of the intervention (0.10 m/s, 95% confidence interval 0.01–0.19, *p* < .05). Sensitivity analysis indicates that larger improvements in walking speed were found over tests covering shorter distances.

**Conclusions:**

Resistance training was found to significantly improve walking speed in people with MS. However, variability in results were noted across studies; accordingly, future research should determine how variables—particularly related to resistance training prescription—influence the intervention effect.

## BACKGROUND

Multiple sclerosis (MS) is a chronic neurological disease characterized by myelin and axonal destruction affecting the central nervous system.^1^ This causes many symptoms including muscular weakness, spasticity, ataxia, fatigue, and balance issues that in combination produce numerous impairments in function.^1^, ^2^ Of these impairments, walking difficulty is one of the most common and debilitating, significantly affecting self‐perceived health, participation in societal roles, and quality of life.^3^, ^4^, ^5^ Walking limitations lead to difficulty completing activities of daily living and impacts employment and socioeconomic status.^4^, ^6^, ^7^, ^8^ Consequently, 70% of people with MS perceive walking limitations as the most debilitating problem associated with MS.^4^

Walking limitations in MS can be characterized by alterations in gait kinematics (ie, altered movement patterns and spatiotemporal parameters),^9^ resulting in approximately 50% of people with MS requiring some form of walking aid within the first 15 years of diagnosis.^3^, ^4^, ^10^ Of these limitations, walking speed—the time taken by an individual to walk a given distance—is consistently found to be reduced in comparison to healthy controls.^9^ Specifically, reductions in walking speed are associated with reduced quality of life and increased disability^11^, ^12^; furthermore, changes in walking speed may even predict disease progression in MS.^13^ Therefore, there is a need for effective interventions to maintain and/or improve walking ability (including speed) in people with MS.

Although the use of disease‐modifying drugs can slow the progression of walking limitations early in the disease course, they do not prevent the onset of disability or reverse existing impairments.^14^ Alternatively, exercise has been suggested as a potential treatment to improve walking ability across the disease‐course of MS.^15^, ^16^ In particular, numerous studies have shown beneficial impacts of resistance training (RT) on lower limb muscular strength and functional capability in persons with MS.^17^, ^18^, ^19^, ^20^, ^21^ Therefore, due to the association between lower limb muscle strength and walking ability in MS, RT may have the potential to improve walking ability in MS populations.^22^

Indeed, current evidence suggests that exercise (including both RT and aerobic exercise) may have a positive impact on walking ability in people with MS.^23^, ^24^, ^25^, ^26^ Considering the relationship between muscle strength and walking ability,^17^, ^18^, ^19^, ^20^, ^21^ there is a need for evidence focusing on RT alone. Additionally, although reviews focusing specifically on RT demonstrate improvements in walking‐related outcomes,^26^ the results do not report the effects of RT specifically for distinct components of overall walking ability (such as walking speed). Therefore, the exact magnitude of changes in walking speed remain unclear; this limits the interpretation and application of findings to clinical practice—particularly when outcomes (such as walking speed) are reported using a standardized scale.^27^ Furthermore, understanding the exact magnitude of change is important for informing the clinical significance of findings using established minimally important difference cutpoints and planning future research through informing sample size calculations. This is of particular relevance considering the importance of walking speed to rehabilitation and the maintenance of functional capacity.^28^

Accordingly, the aim of this systematic review and meta‐analysis was to evaluate the effect of RT on walking speed in people with MS.

## METHODS

This systematic review was based on the Preferred Reporting Items for Systematic Reviews and Meta‐Analyses guidelines for reporting on systematic reviews of randomized controlled trials (RCTs).^29^

### Information sources and search strategies

The following databases were searched from inception to March 16, 2024: CINAHL (via EBSCOhost), MEDLINE (via EBSCOhost), The Allied and Complimentary Medicine Database (via EBSCOhost), Web of Science (via Clarivate), Physiotherapy Evidence Database (PEDro), PsycINFO (via Proquest), and Sports Medicine and Education Index (via ProQuest). Search strategies consisted of a combination of various keywords relating to the following search terms: multiple sclerosis, resistance training, and walking ability. Search strategies from all database searches can be found in Supplementary Material S1.

### Eligibility criteria

To be included in this review, articles must have fulfilled the following: (1) used an RCT design; (2) recruited male or female adults aged 18 years or older with a definite diagnosis of MS regardless of disease duration, phenotype of MS, or disability level; (3) evaluated interventions of any form of RT, with or without supervision, involving the lower extremities regardless of duration of intervention or training session frequency/duration/ intensity/volume; and (4) included at least one objective measure of walking speed.

For the purpose of this review, resistance training was defined as a type of exercise using a variable amount of external resistance performed over one or more sets of a certain number of repetitions.^30^ Walking speed was defined as the time taken by an individual to walk a given distance. Articles were included in this review if the time taken to walk a prespecified distance or the distance walked within a prespecified time were reported, thus allowing walking speed to be calculated.

Studies were excluded based on the following reasons: not published in English, non‐peer‐reviewed publication, nonhuman studies, studies that combined RT with other interventions, and studies including composite measures of overall functional mobility for example timed up and go test, where it was not possible to extract gait speed data.

### Selection process

Results from each database search were exported to Rayyan Intelligent Systematic Review Data Management System (rayyan.qcri.org), where duplicates were removed prior to screening. The titles/abstracts of articles were initially screened by one reviewer (C.M.) based on relevance to the aims of the review as determined by the eligibility criteria. The full texts of remaining articles were then screened for inclusion by two reviewers (C.M., S.R.). Any disagreements were resolved in consultation with a third reviewer (L.P.).

### Methodological quality assessment

The methodological quality of each included article was evaluated using the PEDro scale for RCTs.^31^ The PEDro scale is composed of 11 items and is used to assess an article's risk of bias over the following domains: eligibility criteria, group allocation, blinding, and reporting of outcome measures.^31^ It is frequently used in the assessment of RCT quality in physiotherapy and has acceptable interrater reliability.^32^, ^33^, ^34^ The primary reviewer (C.M.) evaluated all included articles, and scores were compared with an independent secondary reviewer (B.N., L.P., S.R.). No articles were removed based on the assessment of methodological quality. Any disagreements in quality assessment were resolved in consultation with a third reviewer if required.

### Data extraction

Data were extracted by a single reviewer (C.M.) using a standardized data extraction form. The following data items were included for extraction: study characteristics (author[s], date of publication, design, methodological quality), sample characteristics (number recruited and analyzed, gender, age, MS phenotype, level of disability, disease duration), intervention and control characteristics (active/no‐intervention control, form of training, setting, supervision level, intervention/control duration, frequency, intensity, volume, duration of session), walking outcome measures used, and main findings.

### Data synthesis

Data were initially synthesized through narrative synthesis; this consisted of a descriptive overview of key data items extracted from each study alongside the significance and magnitude of changes in walking speed following RT. Subsequently, a meta‐analysis was performed of studies comparing RT with a no‐intervention control using RevMan software (version 5.4) to establish the effect of RT on walking speed. Where studies included data for both an active control and no‐intervention control group, only the no‐intervention control group data were included. Studies that included only comparisons of RT with active control groups were synthesized narratively.

For the purpose of this meta‐analysis, random effects models were used to estimate intervention effect based on the weighted mean difference in walking speed. This was calculated using the mean difference and standard deviation in postintervention walking speed (measured following completion of the intervention period) compared to baseline values for both the intervention and control groups. If the standard deviation for mean difference was not reported in the original article, these missing data were calculated using either (1) 95% confidence intervals (CI) if reported or (2) baseline/postintervention standard deviations according to the Cochrane Handbook for Systematic Reviews guidance.^35^ If any article did not report the mean difference and standard deviation for the intervention and control groups, or did not provide sufficient data for the result to be calculated, it was excluded from the meta‐analysis. For all tests, a significance level of *p* < .05 was used.

In order to prepare data for meta‐analysis, results in which the distance walked over a prespecified time (eg, 6‐minute Walk Test) or the time taken to walk a prespecified distance (eg, 10‐m Walk Test) were converted to walking speed. For consistency, all units of walking speed were converted to m/s. Results from studies that included multiple walking tests were combined to create a single pairwise comparisons for the purposes of the main meta‐analysis.^35^ However, due to the heterogeneity in outcome measured used, sensitivity analyses were also used to stratify results based on long walking tests (2‐Minute Walk Test [2MWT]/6‐Minute Walk Test [6MWT]) and short walking tests (10‐Meter Walk Test [10MWT], Timed 25‐Foot Walk Test [T25FWT], 50‐Meter Walk Test [50MWT]). Due to the availability of data from the included studies, all data relate to the maximum walking speeds recorded.

## RESULTS

### Search results

**FIGURE 1:**
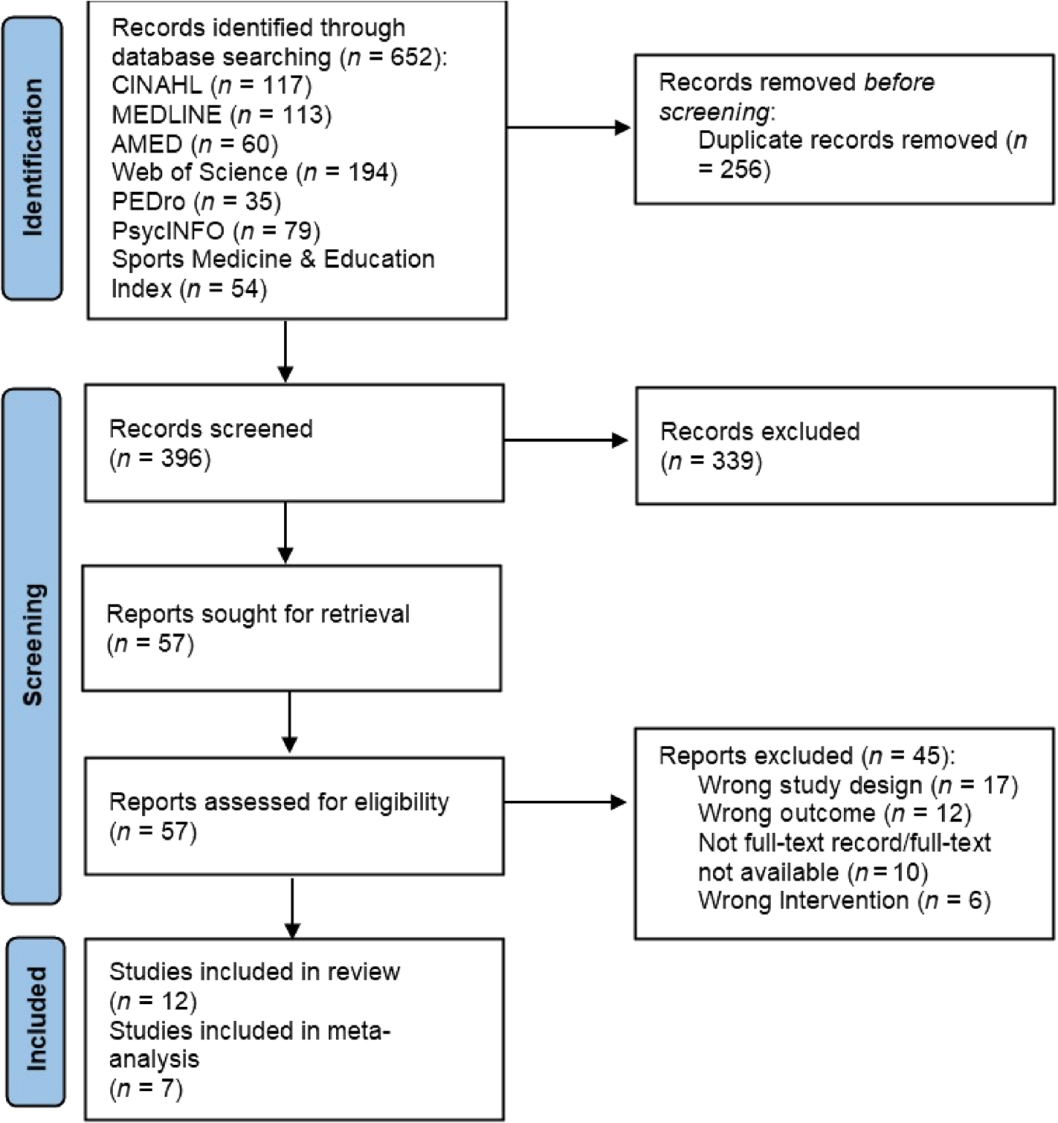
Preferred Reporting Items for Systematic Reviews and Meta‐Analyses flow diagram.^27^ PEDro, Physiotherapy Evidence Database.

Searching the chosen electronic databases identified 652 potential articles for inclusion. Following the removal of duplicates, the titles and abstracts of 396 articles were screened and 339 articles were excluded (Figure 1). The remaining 57 articles were sought for retrieval for full‐text screening. Of those, 45 articles were excluded; 17 excluded for using non‐RCT study designs, 12 excluded for using non‐walking‐specific outcome measures, 10 excluded for not being peer‐reviewed publication/full‐text availability, and 6 excluded for not including RT involving the lower extremities. As a result, 12 articles were included in the review (Table 1).^36^, ^37^, ^38^, ^39^, ^40^, ^41^, ^42^, ^43^, ^44^, ^45^, ^46^, ^47^

**TABLE 1:** Characteristics of included articles.

Study; design; quality	Intervention/control description	Sample details per group	Intervention delivery mode; duration/frequency; frequency; intensity; volume; session duration	Outcome measure; time points	Main intragroup effect^a^	Main intergroup effect
Harvey et al.^36^; RCT; PEDro = 5	Int = weighted leg extension exercise	Recruited: *N* = 7 Analyzed: *N* = 6 Gender (F, M): 5 F, 1 M Age (y) (mean): 38.0 MS type (n): RR = 6 EDSS: NR MS duration (y) (range) = 2.5–20	Mode: individual/unsupervised Duration/frequency: 8 weeks, 2x/day, 7 days/week Intensity: 0.5 kg or 1.0 kg ankle weights Volume: 5 sets of 10 repetitions Session duration: NR Muscle group(s) Targeted: knee extensors	10MWT, 50MWT; 0, 8 weeks	10MWT (m/s): 0 weeks: 1.14 (SD NR); 8 weeks: 1.25 (SD NR), *p* > .05 50MWT (m/s): 0 weeks: 1.08 (SD NR); 8 weeks: 1.09 (SD NR), *p* > .05	NR
	AC = mobility exercise	Recruited: *N* = 7 Analyzed: *N* = 6 Gender (F, M): 5 F, 1 M Age (y) (mean): 49.0 MS type (n): RR = 6 EDSS: NR MS duration (y) (range) = 1–15	Mode: individual/unsupervised Duration/frequency: 8 weeks, 7 days/week Intensity: N/A Volume: NR Session duration: NR Muscle group(s) targeted: N/A	10MWT, 50MWT; 0, 8 weeks	10MWT (m/s): 0 weeks: 1.37 (SD NR); 8 weeks: 1.46 (SD NR), *p* > .05 50MWT (m/s): 0 weeks: 1.22 (SD NR); 8 weeks: 1.25 (SD NR), *p* > .05	
	NIC = usual activity	Recruited: *N* = 5 Analyzed: *N* = 5 Gender (F, M): 4 F, 1 M Age (y) (mean): 43.0 MS type (n): RR = 6 EDSS: NR MS duration (y) (range) = 1.5–8	Mode: individual/unsupervised Duration/frequency: 8 weeks Intensity: N/A Volume: N/A Session duration: N/A Muscle group(s) targeted: N/A	10MWT, 50MWT; 0, 8 weeks	10MWT (m/s): 0 weeks: 1.35 (SD NR); 8 weeks: 1.25 (SD NR), *p* > .05 50MWT (m/s): 0 weeks: 1.26 (SD NR); 8 weeks: 1.17 (SD NR), *p* > .05	
Dalgas et al.^37^; RCT; PEDro = 4	Int = lower body RT	Recruited: *N* = 19 Analyzed: *N* = 15 Gender (F, M): 10 F, 5 M Age (y) (mean): 47.7 (95% CI 41.9–53.4) MS type (n): RR = 15 EDSS (mean): 3.7 (95% CI 3.2–4.2) MS duration (y) (mean) = 6.6 (95% CI 3.3–9.8)	Mode: group/supervised Duration/frequency: 12 weeks, 2 days/week Intensity: 8–15 RM Volume: 3–4 sets of 10–12 repetitions Session duration: NR Muscle group(s) Targeted: knee extensors, knee flexor, hip extensors, hip flexors	10MWT, 6MWT; 0, 12, 24 weeks	10MWT (m/s) 0 weeks: 1.29 (95% CI 1.03–1.79); 12 weeks: 1.52 (95% CI 1.19–2.04), *p* < .05; 24 weeks: 1.49 (95% CI 1.22–1.96), *p* < .05 6MWT (m/s) 0 weeks: 1.22 (95% CI 0.96–1.49); 12 weeks: 1.38 (95% CI 1.11–1.64), *p* < .05; 24 weeks: 1.37 (95% CI 1.10–1.64), *p* < .05	NR
	NIC = Usual activity	Recruited: *N* = 19 Analyzed: *N* = 16 Gender (F, M): 10 F, 6 M Age (y) (mean): 49.1 (95% CI 44.6–53.6) MS type (n): RR = 16 EDSS (mean): 3.9 (95% CI 3.5–4.4) MS duration (y) (mean) = 8.1 (95% CI 4.9–11.3)	Mode: Individual/Unsupervised Duration/Frequency: 12 weeks Intensity: N/A Volume: N/A Session Duration: N/A Muscle Group(s) Targeted: N/A		10MWT (m/s) 0 weeks: 1.37 (95% CI 1.16–1.69); 12 weeks: 1.27 (95% CI 1.01–1.67), *p* < .05; 24 weeks: 1.32 (95% CI 1.03–1.85), *p* > .05 6MWT (m/s) 0 weeks: 1.22 (95% CI 1.02–1.41); 12 weeks: 1.21 (95% CI 0.99–1.44), *p* > .05; 24 weeks: 1.27 (95% CI 1.02–1.51), *p* > .05	
Broekmans et al.^38^; RCT; PEDro = 4	Int = Lower Body RT	Recruited: *N* = 11 Analyzed: *N* = 11 Gender (F, M): 6 F, 5 M Age (y) (mean ± SD): 44.9 ± 11.6 MS type (n): RR = 5, SP = 3, PP = 3 EDSS (mean ± SD): 4.5 ± 1.3 MS duration (y) = NR	Mode: NR Duration/frequency: 20 weeks, 5 days/2 weeks Intensity: 50–60% 1RM; 10‐15RM Volume: 1–2 set of 10–15 repetitions Session duration: 60 min Muscle group(s) targeted: hip extensors, knee extensors, knee flexors	T25FW, 2MWT; 0, 10, 20 weeks	T25FW (m/s) 0 weeks: 1.23 ± 0.14; 10 weeks: *p* > .05; 20 weeks: *p* > .05 2MWT (m/s): 0 weeks: 1.39 ± 0.07; 10 weeks: *p* > .05; 20 weeks: *p* > .05	NR
	AC = lower body RT + simultaneous muscle electro‐stimulation	Recruited: *N* = 11 Analyzed: *N* = 10 Gender (F, M): 6 F, 5 M Age (y) (mean ± SD): 48.7 ± 8.6 MS type (n): RR = 2, SP = 4, PP = 4 EDSS (mean ± SD): 4.4 ± 0.9 MS duration (y) = NR	Mode: NR Duration/frequency: 20 weeks, 5 days/2 weeks Intensity: 50–60% 1 RM; 10–15 RM Volume: 1–2 set of 10–15 repetitions Session duration: 60 min Muscle group(s) targeted: hip extensors, knee extensors, knee flexors	T25FW, 2MWT; 0, 10, 20 weeks	T25FW (m/s): 0 weeks: 1.41 ± 0.10; 10 weeks: *p* > .05; 20 weeks: *p* > .05 2MWT (m/s): 0 weeks: 1.49 ± 0.11; 10 weeks: *p* > .05; 20 weeks: *p* > .05	
	NIC = usual activity	Recruited: *N* = 14 Analyzed: *N* = 12 Gender (F, M): 11 F, 3 M Age (y) (mean ± SD): 49.7 ± 11.3 MS type (n): RR = 6, SP = 3, PP = 3 EDSS (mean ± SD): 4.1 ± 1.1 MS duration (y) = NR	Mode: NR Duration/frequency: 20 weeks Intensity: N/A Volume: N/A Session duration: N/A Muscle group(s) targeted: N/A	T25FW, 2MWT; 0, 10, 20 weeks	T25FW (m/s): 0 weeks: 1.31 ± 0.09; 10 weeks: *p* > .05; 20 weeks: *p* > .05 2MWT (m/s): 0 weeks: 1.38 ± 0.06; 10 weeks: *p* > .05; 20 weeks: *p* > .05	
Dodd et al.^39^; RCT; PEDro = 8	Int = lower body RT	Recruited: *N* = 39 Analyzed: *N* = 36 Gender (F, M): 26 F, 10 M Age (y) (mean ± SD): 47.7 ± 10.8 MS type (n): RR = 36 EDSS: NR MS duration (y) = NR	Mode: group/supervised Duration/fgrequency: 10 weeks, 2 days/week Intensity: 10–12 RM Volume: 2 sets of 10–12 repetitions Session duration: 45 min Muscle group(s) targeted: hip extensors, hip flexors, knee extensors, knee flexors, ankle dorsiflexors, ankle plantar flexors	2MWT; 0, 10, 22 weeks	2MWT (m/s): 0 weeks: 1.00 ± 0.30; 10 weeks: 1.02 ± 0.29, *p* > .05; 22 weeks: 0.99 ± 0.33, *p* > .05	Effect Size (d) (95% CI): Week 0–1: d = 0.27 (−0.20–0.74), *p* > .05 Week 0–22: d = 0.12 (−0.34–0.59), *p* > .05
	NIC = usual activity	Recruited: *N* = 37 Analyzed: *N* = 35 Gender (F, M): 26 F, 9 M Age (y) (mean ± SD): 50.4 ± 9.6 MS type (n): RR = 35 EDSS: NR MS duration (y) = NR	Mode: N/A Duration/frequency: 10 weeks Intensity: N/A Volume: N/A Session duration: N/A Muscle group(s) targeted: N/A	2MWT; 0, 10, 22 weeks	2MWT (m/s): 0 weeks: 0.93 ± 0.31; 10 weeks: 0.94 ± 0.32, *p* > .05; 22 weeks: 0.95 ± 0.34, *p* > .05	
Sabapathy et al.^40^; randomized crossover pilot study; PEDro = 3	Int = upper body/lower body, core, stability RT	Whole sample: Recruited: *N* = 21 Analyzed: *N* = 16 Gender (F, M): 12 F, 4 M Age (y) (mean ± SD): 55.0 ± 7.0 MS type (n): RR = 10, SP = 3, PP = 3 EDSS: NR MS duration (y) (mean ± SD) = 10 ± 10	Mode: supervised Duration/frequency: 8 weeks, 2 days/week Intensity: 3–5 on Borg Scale Volume: 2–3 sets of 6–10 repetitions Session duration: NR Muscle group(s) targeted: scapula retractors, shoulder adductors, shoulder abductors, core, hip extensors, hip abductors, knee extensors, knee flexors, ankle plantar flexors, ankle dorsiflexors	6MWT; 0, 8 weeks	6MWT (m/s): 0 weeks: 1.24 ± 0.31; 8 weeks: 1.35 ± 0.30, *p* < .05	6MWT: *p* > .05
	AC = circuit endurance training		Mode: supervised Duration/frequency: 8 weeks, 2 days/week Intensity: 3–5 on Borg RPE Scale Volume: 5 mins of work per exercise station (8 stations) Session duration: 48 mins Muscle group(s) targeted: hip extensors, knee extensors, knee flexors, shoulder flexors, shoulder extensors, elbow flexors, elbow extensors	6MWT; 0, 8 weeks	6MWT (m/s): 0 weeks: 1.34 ± 0.27; 8 weeks: 1.40 ± 0.28, *p* < .05	
Kjolhede et al.^41^; RCT; PEDro = 7	Int = upper/lower body RT	Recruited: *N* = 18 Analyzed: *N* = 17 Gender (F, M): NR Age (y): NR MS type (n): RR = 17 EDSS: NR MS duration (y): NR	Mode: supervised Duration/frequency: 24 weeks, 2 days/week Intensity: 6–15 RM Volume: 3–5 sets of 6–12 repetitions Session duration: NR Muscle group(s) targeted: hip extensors, hip flexors, knee extensors, knee flexors, ankle plantar flexors, shoulder extensors, elbow extensors	T25FW, 2MWT; 0, 24, 48 weeks	T25FW (m/s): 0 weeks: 1.65 ± 0.1; 24 weeks: 1.82 ± 0.1, *p* < .05; 48 weeks: 1.80 ± 0.1, *p* < .05 2MWT (m/s): 0 weeks: 1.60 ± 0.1; 24 weeks: 1.78 ± 0.1, *p* < .05; 48 weeks: 1.75 ± 0.1, *p* < .05	Mean difference (95% CI): Week 0–24: T25FW: −0.16 (−0.27 to 0.05); 2MWT: −0.14 (−0.25 to −0.03) Week 0–48: T25FW: −0.11 (−0.23 to 0.05); 2MWT: −0.05 (−0.17 to 0.06)
	NIC = waitlist control (usual activity)	Recruited: *N* = 17 Analyzed: *N* = 15 Gender (F, M): NR Age (y): NR MS type (n): RR = 15 EDSS: NR MS duration (y): NR	Mode: N/A Duration/frequency: 24 weeks Intensity: N/A Volume: N/A Session duration: N/A Muscle group(s) targeted: N/A	T25FW, 2MWT; 0, 24, 48 weeks	T25FW (m/s): 0 weeks: 1.77 ± 0.1; 24 weeks: 1.77 ± 0.1, *p* > .05; 48 weeks: 1.86 ± 0.1, *p* < .05 2MWT (m/s): 0 weeks: 1.63 ± 0.1; 24 weeks: 1.67 ± 0.1, *p* > .05; 48 weeks: 1.69 ± 0.1, *p* > .05	
Moradi et al.^42^; RCT; PEDro = 5	Int = upper/lower body RT program	Recruited: *N* = 10 Analyzed: *N* = 8 Gender (F, M): 0 F, 8 M Age (y) (mean ± SD): 34.4 ± 11.07 MS type (n): RR = 5, SP = 3 EDSS (median): 3 (range: 1–6) MS duration (y) (mean ± SD) = 8.1 ± 4.8	Mode: NR Duration/frequency: 8 weeks, 3 days/week Intensity: 50–80% of 1 RM Volume: 1 set of 6–15 repetitions Session duration: 30 min Muscle group(s) targeted: scapula retractors, shoulder adductors, hip extensors, knee extensors	10MWT; 0, 8 weeks	10MWT (m/s): 0 weeks: 1.20 ± 0.27; 8 weeks: 1.52 ± 0.76, *p* < .05	10MWT: *p* > .05
	NIC = usual activity	Recruited: *N* = 10 Analyzed: *N* = 10 Gender (F, M): 0 F, 10 M Age (y) (mean ± SD): 33.1 ± 7.08 MS type (n): RR = 6, SP = 4 EDSS (median): 3 (range: 1–5) MS duration (y) (mean ± SD) = 6.5 ± 5.8	Mode: NR Duration/frequency: 8 weeks Intensity: N/A Volume: N/A Session duration: N/A Muscle group(s) targeted: N/A	10MWT; 0, 8 weeks	10MWT (m/s): 0 weeks: 1.34 ± 0.29; 8 weeks: 1.35 ± 0.29, *p* > .05	
Manca et al.^43^; RCT; PEDro = 8	Int = high‐intensity concentric resistance training of less affected (stronger) ankle dorsiflexor	Recruited: *N* = 15 Analyzed: *N* = 13 Gender (F, M): 11 F, 4 M Age (y) (mean ± SD): 42.9 ± 14.7 MS type (n): RR = 15 EDSS (mean ± SD): 3.8 ± 1.39 MS duration (y) (mean ± SD) = 12.7 ± 10.1	Mode: NR Duration/frequency: 6 weeks, 3 days/week Intensity:45 degrees per second of angular velocity and 10 degrees per second of angular velocity Volume: 3 sets of 4 repetitions at each intensity Session duration: 25 min Muscle group(s) targeted: ankle dorsiflexors	10MWT, 2MWT, 6MWT; 0, 6, 12 weeks	10MWT (m/s): 0 weeks: 1.08 ± 0.35; 6 weeks: 1.16 ± 0.39, *p* < .05; 12 weeks: 1.09 ± 0.34, *p* > .05 2MWT (m/s): 0 weeks: 0.99 ± 0.24; 6 weeks: 1.10 ± 0.26, *p* < .05; 12 weeks: 1.01 ± 0.22, *p* > .05 6MWT (m/s): 0 weeks: 0.92 ± 0.27; 6 weeks: 1.01 ± 0.27, *p* < .05; 12 weeks: 0.96 ± 0.27, *p* > .05	10MWT: *p* > .05 2MWT: *p* > .05 6MWT: *p* > .05
	AC = high‐intensity concentric resistance training of more affected (weaker) ankle dorsiflexor	Recruited: *N* = 15 Analyzed: *N* = 13 Gender (F, M): 13 F, 2 M Age (y) (mean ± SD): 47.3 ± 9.4 MS type (n): RR = 15 EDSS (mean ± SD): 3.0 ± 1.00 MS duration (y) (mean ± SD) = 16.8 ± 8.3	Mode: NR Duration/frequency: 6 weeks, 3 days/week Intensity:45 degrees per second of angular velocity and 10 degrees per second of angular velocity Volume: 3 sets of 4 repetitions at each intensity Session duration: 25 min Muscle group(s) targeted: ankle dorsiflexors	10MWT, 2MWT, 6MWT; 0, 6, 12 weeks	10MWT (m/s): 0 weeks: 1.15 ± 0.13; 6 weeks: 1.28 ± 0.16, *p* < .05; 12 weeks: 1.16 ± 0.12, *p* > .05 2MWT (m/s): 0 weeks: 1.09 ± 0.22; 6 weeks: 1.21 ± 0.17, *p* < .05; 12 weeks: 1.12 ± 0.20, *p* > .05 6MWT (m/s): 0 weeks: 1.02 ± 0.22; 6 weeks: 1.09 ± 0.20, *p* < .05; 12 weeks: 1.06 ± 0.21, *p* > .05	
Hosseini et al.^44^; RCT; PEDro = 5	Int = home‐based lower body RT	Recruited: *N* = 9 Analyzed: *N* = 8 Gender (F, M): 5 F, 4 M Age (y) (mean ± SD): 32.9 ± 8.13 MS type (N): NR EDSS: range = 1–6 MS duration (y) = NR	Mode: individual/unsupervised Duration/frequency: 8 weeks, 3 days/week Intensity: 1% of body weight with 0.5–1% of body weight added every 2 weeks Volume: 3 sets of 10 repetitions Session duration: 25–30 min Muscle group(s) targeted: hip extensors, knee flexors, knee extensors, ankle plantar flexors	10MWT; 0, 8 weeks	10MWT (m/s): 0 weeks: 1.47 ± 0.28; 8 weeks: 1.49 ± 0.25, *p* > .05	10MWT: *p* > .05
	AC = hatha yoga	Recruited: *N* = 9 Analyzed: *N* = 8 Gender (F, M): 5 F, 4 M Age (y) (mean ± SD): 31.3 ± 7.09 MS type (N): NR EDSS: range = 1–6 MS duration (y) = NR	Mode: individual/unsupervised Duration/frequency: 8 weeks, 3 days/week Intensity: N/A Volume: 10–30s per pose Session duration: 60–70 min Muscle group(s) targeted: N/A	10MWT; 0, 8 weeks	10MWT (m/s): 0 weeks: 1.72 ± 0.25; 8 weeks: 1.75 ± 0.27, *p* > .05	
	NIC = usual activity	Recruited: *N* = 8 Analyzed: *N* = 8 Gender (F, M): 4 F, 4 M Age (y) (mean ± SD): 33.0 ± 9.74 MS type (N): NR EDSS: range = 1–6 MS duration (y) = NR	Mode: individual/unsupervised Duration/frequency: 8 weeks Intensity: N/A Volume: N/A Session duration: N/A Muscle group(s) targeted: N/A	10MWT; 0, 8 weeks	10MWT (m/s): 0 weeks: 1.52 ± 0.33; 8 weeks: 1.52 ± 0.39, *p* > .05	
Callesen et al.^45^; RCT; PEDro = 7	Int = lower body progressive RT	Recruited: *N* = 23 Analyzed: *N* = 17 Gender (F, M): 16 F, 7 M Age (y) (median): 52 (IQR: 47–59) MS type (n): RR = 16. SP = 5, PP = 2 EDSS: median = 4 MS duration (y) (median): 15 (IQR: 8–17)	Mode: supervised Duration/frequency: 10 weeks, 2 days/week Intensity: 8–15 RM Volume: 3–4 sets of 8–12 repetitions Session duration: 60 min Muscle group(s) targeted: hip extensors, hip flexors, knee extensors, knee flexors, ankle plantar flexors	T25FW, 6MWT; 0, 10 weeks	T25FW (m/s): 0 weeks: 1.24 ± 0.34; mean change postintervention (95% CI): 0.06 (−0.01 to 0.13), *p* > .05 6MWT (m/s): 0 weeks: 1.06 ± 0.34; mean change postintervention (95% CI): 0.06 (0.01 to 0.11), *p* < .05	T25FW: AC vs NIC: *p* < .05 Int vs NIC: *p* > .05 AC vs Int: *p* > .05 6MWT: AC vs NIC: *p* > .05 Int vs NIC: *p* > .05 AC vs Int: *p* > .05
	AC = Balance and motor control training	Recruited: *N* = 28 Analyzed: *N* = 24 Gender (F, M): 23 F, 5 M Age (y) (median): 51 (IQR: 43–56) MS type (n): RR = 21, SP = 4, PP = 3 EDSS: median = 4 MS duration (y) (median): 10 (IQR: 5–20)	Mode: supervised Duration/frequency: 10 weeks, 2 days/week Intensity: NR Volume: N/A Session duration: 60 min Muscle group(s) targeted: N/A	T25FW, 6MWT; 0, 10 weeks	T25FW (m/s): 0 weeks: 1.29 ± 0.30; mean change postintervention (95% CI): 0.14 (0.08 to 0.20), *p* < .05 6MWT (m/s): 0 weeks: 1.08 ± 0.32; change postintervention (95% CI): 0.08 (0.04 to 0.12), *p* < .05	
	NIC = waitlist control (usual activity)	Recruited: *N* = 20 Analyzed: *N* = 18 Gender (F, M): 16 F, 4 M Age (y) (median): 56 (IQR: 50–59) MS type (n): RR = 13, SP = 3, PP = 4 EDSS: median = 3.5 MS duration (y) (median): 11 (IQR: 4–18)	Mode: N/A Duration/frequency: 10 weeks Intensity: N/A Volume: N/A Session duration: N/A Muscle group(s) targeted: N/A	T25FW, 6MWT; 0, 10 weeks	T25FW (m/s): 0 weeks: 1.22 ± 0.40; mean change postintervention (95% CI): 0.04 (−0.03 to 0.11), *p* > .05 6MWT (m/s): 0 weeks: 1.08 ± 0.34; change postintervention (95% CI): 0.03 (−0.02 to 0.08), *p* > .05	
Andreu‐Caravaca et al.^46^; RCT; PEDro = 7	Int = fast‐velocity concentric RT	Recruited: *N* = 18 Analyzed: *N* = 18 Gender (F, M): 8 F, 10 M Age (y) (mean ± SD): 44.9 ± 10.6 MS type (n): RR = 16, SP = 2 EDSS (mean ± SD) = 3.2 ± 1.7 MS duration (y) = NR	Mode: group/supervised Duration/frequency: 10 weeks, 3 days/week Intensity (% 1‐RM): 60–75% of 1RM Volume: 2–4 sets of 8–15 repetitions Session duration: NR Muscle group(s) targeted: hip extensors, knee extensors, knee flexors, ankle plantar flexors	10MWT, 6MWT; 0, 10 weeks	10MWT (m/s): 0 weeks: 2.2 ± 1.5; 10 weeks: 2.6 ± 1.9, *p* < .05 6MWT (m/s): 0 weeks: 1.24 ± 0.61; 10 weeks: 1.55 ± 0.66, *p* < .05	10MWT: *p* > .05 6MWT: *p* > .05
	NIC = usual activity	Recruited: *N* = 12 Analyzed: *N* = 12 Gender (F, M): 7 F, 5 M Age (y) (mean ± SD): 48.4 ± 10.2 MS type (n): RR = 11, SP = 1 EDSS (mean ± SD) = 3.3 ± 1.3 MS duration (y) = NR	Mode: N/A Duration/frequency: 10 weeks Intensity: N/A Volume: N/A Session duration: N/A Muscle group(s) targeted: N/A	10MWT, 6MWT; 0, 10 weeks	10MWT (m/s): 0 weeks: 1.1 ± 1.0; 10 weeks: 1.25 ± 1.1, *p* > .05 6MWT (m/s): 0 weeks: 0.99 ± 0.69; 10 weeks: 1.09 ± 0.86, *p* > .05	
Ghahfarrokhi et al^47^; RCT pilot study; PEDro = 8	Int = home‐based upper/lower body, core RT	Recruited: *N* = 15 Analyzed: *N* = 15 Gender (F, M): 12 F, 3 M Age (y) (mean ± SD): 39.9 ± 9.09 MS type (n): RR = 15 EDSS (mean ± SD) = 4.3 ± 0.97 MS duration (y) (mean ± SD) = 9.73 ± 5.29	Mode: individual/unsupervised Duration/frequency: 8 weeks, 3 days/week Intensity: 2–6 on Borg RPE Scale Volume: 1–2 sets of 10–12 repetitions Session duration: 120–190 min Muscle group(s) targeted: shoulder flexors, shoulder extensors, elbow flexors, elbow extensors, core, hip flexors, hip extensors, knee extensors, knee flexors, ankle plantar flexors, ankle dorsiflexors	6MWT, 10MWT, T25FW; 0, 8 weeks	6MWT (m/s): 0 weeks: 0.72 ± 0.07; 8 weeks: 0.74 ± 0.10, *p* < .05 10MWT (m/s): 0 weeks: 0.32 ± 0.19; 8 weeks: 0.41 ± 0.18, *p* > .05 T25FW (m/s): 0 weeks: 0.74 ± 0.64; 8 weeks: 0.69 ± 0.26, *p* > .05	6MWT: *p* < .05 10MWT: *p* > .05 T25FWT: *p* > .05
	AC = home‐based neurofunctional training	Recruited: *N* = 15 Analyzed: *N* = 15 Gender (F, M): 13 F, 2 M Age (y) (mean ± SD): 37.5 ± 8.58 MS type (n): RR = 15 EDSS (mean ± SD) = 4.57 ± 1.3 MS duration (y) (mean ± SD) = 8.3 ± 6.48	Mode: individual/unsupervised Duration/frequency: 8 weeks, 3 days/week Intensity: 2–6 on Borg RPE Scale Volume: 2 sets of varying repetitions per exercise Session duration: 90–120 min Muscle group(s) targeted: N/A	6MWT, 10MWT, T25FW; 0, 8 weeks	6MWT (m/s): 0 weeks: 0.69 ± 0.07; 8 weeks: 0.81 ± 0.11, *p* < .05 10MWT (s); (m/s): 0 weeks: 0.79 ± 0.44; 8 weeks: 0.93 ± 0.39, *p* > .05 T25FW (m/s): 0 weeks: 0.86 ± 0.60; 8 weeks: 0.96 ± 0.33, *p* > .05	

Abbreviations: AC, active control; CI, confidence interval; EDSS, Expanded Disability Status Scale; F, female; Int, intervention; IQR, interquartile range; M, Male; MS, multiple sclerosis; N, number; N/A, not applicable; NIC, no‐intervention control; NR, not reported; PEDro, Physiotherapy Evidence Database; PP, primary progressive; RCT, randomized controlled trial; RM, repetition maximum; RPE, rate of perceived exertion; RR, relapsing‐remitting; RT, resistance training; SP, secondary progressive; T25FW, Timed 25‐Foot Walk; y, years; 2MWT, 2‐Minute Walk Test; 6MWT, 6‐Minute Walk Test; 10MWT, 10‐Meter Walk Test; 50MWT, 50‐Meter Walk Test.

^a^ Descriptive baseline and final values presented as mean ± SD unless stated otherwise.

### Study Design

All 12 of the included articles were RCTs; two of which were pilot RCTs,^40^, ^45^ including one that was a randomized cross over trial design.^40^ Eight of the studies included two trial arms (intervention and either active or no‐intervention control),^37^, ^39^, ^40^, ^41^, ^42^, ^43^, ^46^, ^47^ whereas four of the articles included three trial arms (intervention, active control, and no‐intervention control).^36^, ^38^, ^44^, ^45^ The duration of intervention ranged from 6 to 24 weeks, with 8 weeks as the most commonly reported intervention length.^36^, ^40^, ^41^, ^42^, ^44^

### Participant Characteristics

A total of 425 participants were included across all 12 studies, with sample sizes ranging from 19 to 76 participants. Of those, 180 participants received an RT intervention, 85 received an active control, and 139 were assigned to a no‐intervention control condition. One cross‐over study had all 21 participants receive both an intervention and active control condition in alternating periods.^40^ Disability, as assessed by the Expanded Disability Status Scale, across all studies ranged from 1.0 to 6.0—indicating mild to moderate disability. Of those studies who reported MS phenotypes, 322 had relapsing‐remitting (RR), 35 had secondary progressive, and 22 had primary progressive. Six studies recruited RR MS participants only.^36^, ^37^, ^39^, ^41^, ^43^, ^47^ Disease duration of participants across all studies ranged from 5 to 17 years.

### Outcome measures

Across all studies, there were five objective walking outcome measures used: 10MWT, T25FWT, 50MWT, 2MWT, and 6MWT. The 10MWT was the most commonly used (*n* = 7),^36^, ^37^, ^42^, ^43^, ^44^, ^46^, ^47^ four studies used the T25FWT^38^, ^41^, ^45^, ^47^ and 2MWT,^38^, ^39^, ^41^, ^43^ whereas five studies used the 6MWT.^37^, ^40^, ^43^, ^45^, ^47^ Only one study used the 50MWT.^36^ All 12 studies conducted baseline and postintervention measurements,^36^, ^37^, ^38^, ^39^, ^40^, ^41^, ^42^, ^43^, ^44^, ^45^, ^46^, ^47^ and one study also performed midintervention measurements.^38^ Four studies also conducted follow‐up outcome measurements: 6 weeks,^43^ 12 weeks,^37^, ^39^ and 24 weeks^41^ postintervention. Tests were performed at maximum walking speed and at baseline the mean walking speed ranged from 0.32 to 2.20 m/s in the intervention groups and 0.69 to 1.77 m/s in the control groups.

### Intervention characteristics

In line with the review eligibility criteria, RT interventions were delivered across all 12 studies.^36^, ^37^, ^38^, ^39^, ^40^, ^41^, ^42^, ^43^, ^44^, ^45^, ^46^, ^47^ Seven articles included interventions of RT solely of lower body exercises.^36^, ^37^, ^38^, ^39^, ^44^, ^45^, ^46^ Four articles provided RT programs involving both upper and lower body exercises,^40^, ^41^, ^42^, ^47^ of which two studies also included core exercises.^40^, ^47^ One study compared a high intensity concentric RT program of the less affected/stronger ankle dorsiflexor muscles to one training the more affected/weaker ankle dorsiflexor muscles.^43^ Changes in muscle strength were measured in 10 studies,^36^, ^37^, ^38^, ^39^, ^41^, ^42^, ^43^, ^44^, ^45^, ^46^ with nine reporting a statistically significant change in strength following the RT interventions.^37^, ^38^, ^39^, ^41^, ^42^, ^43^, ^44^, ^45^, ^46^

Of those articles reporting mode of delivery, six of the interventions were supervised,^37^, ^39^, ^40^, ^41^, ^45^, ^46^ and three were unsupervised^36^, ^44^, ^47^; three interventions were performed individually,^36^, ^44^, ^47^ and three were provided in a group setting.^37^, ^39^, ^46^ Regarding frequency of training sessions, the most commonly reported were 3 days per week,^42^, ^43^, ^44^, ^46^, ^47^ and 2 days per week.^37^, ^39^, ^40^, ^41^, ^45^ One study prescribed a single exercise, which was performed twice each day.^36^ Another article structured sessions 2 days every 2 weeks^38^ but also had a longer intervention duration (20 weeks) compared to other studies.

The majority of articles determined intervention intensity based on the concept of repetition maximum, which was progressed throughout the intervention duration (*n* = 7).^37^, ^38^, ^39^, ^41^, ^42^, ^45^, ^46^ Two articles used the Borg Rating of Perceived Exertion Scale to establish intensity, of which one progressed intensity over the intervention duration^47^ and one kept the same intensity for the entire intervention.^40^

Regarding training volume per session (sets/repetitions); five of the studies kept a consistent volume throughout the intervention,^36^, ^39^, ^40^, ^43^, ^44^ whereas the other seven varied volume throughout the intervention in accordance with progression of intensity.^37^, ^38^, ^41^, ^42^, ^45^, ^46^, ^47^ Of those studies reporting training session duration, the most common were 25 to 30 minutes^42^, ^43^, ^44^ and 60 minutes.^38^, ^45^ Only one article involved session duration longer than 60 minutes.^47^

Of the studies that reported the form of resistance, the most commonly used was weight machines (*n* = 5).^38^, ^39^, ^42^, ^45^, ^46^ Two studies used weights attached to the body,^36^, ^44^ and one study used resistance bands.^47^ One study used an isokinetic dynamometer device, which was also used for strength testing.^43^ Another study used a variety of resistance types including bodyweight, dumbbells, resistance bands, and weights attached to the body.^40^ The most commonly targeted muscle groups were knee extensors (*n* = 11),^36^, ^37^, ^38^, ^39^, ^40^, ^41^, ^42^, ^44^, ^45^, ^46^, ^47^ knee flexors (*n* = 9),^37^, ^38^, ^39^, ^40^, ^41^, ^44^, ^45^, ^46^, ^47^ hip extensors (*n* = 9),^38^, ^39^, ^41^, ^42^, ^44^, ^45^, ^46^, ^47^ and ankle plantar flexors (*n* = 7).^39^, ^40^, ^41^, ^44^, ^45^, ^46^, ^47^

### Control group characteristics

Across all articles, three studies used active control groups,^40^, ^43^, ^47^ five implemented solely no‐intervention control groups,^37^, ^39^, ^41^, ^42^, ^46^ and four used both active and no‐intervention control groups.^36^, ^38^, ^44^, ^45^ Active control interventions were delivered in the forms of: hatha yoga,^44^ mobility exercise,^36^ home‐based neurofunctional training,^47^ balance and motor control training,^45^ and circuit endurance training.^40^ One article compared RT alone with RT plus simultaneous muscle electro‐stimulation (which was the primary intervention of this study),^38^ and another compared two forms of ankle dorsiflexor muscle RT.^43^ All of the studies implementing no‐intervention control groups used “usual activity.”^36^, ^37^, ^38^, ^39^, ^41^, ^42^, ^44^, ^45^, ^46^

### Quality assessment

The total scores of quality assessment on the PEDro scale for RCTs ranged from 3 to 8 (Table 2). No articles were excluded from the review based on their quality assessment score. Articles tended to score lower due to lack of concealment during group allocation^38^, ^40^, ^42^, ^44^, ^46^ and not reporting between‐group results, significantly affecting the ability to conclude on intervention effectiveness.^36^, ^37^, ^38^, ^40^ None of the studies were able to blind participants to the treatment they were given, based on the nature of the exercise interventions. With the exception of two studies,^36^, ^40^ most groups were similar at baseline, reducing the effect of any potential confounding variables on study results.

**TABLE 2:** PEDro scale for included articles.

Article	PEDro Scale Item^a^											
	1	2	3	4	5	6	7	8	9	10	11	Total (0–10)
Harvey et al.^36^	1	1	1	0	0	0	0	1	1	0	1	5
Dalgas et al.^37^	1	1	1	1	0	0	0	0	0	0	1	4
Broekmans et al.^38^	1	1	0	1	0	0	0	1	0	0	1	4
Dodd et al.^39^	1	1	1	1	0	0	1	1	1	1	1	8
Sabapathy et al.^40^	1	1	0	0	0	0	0	1	0	0	1	3
Kjolhede et al.^41^	1	1	1	1	0	0	0	1	1	1	1	7
Moradi et al.^42^	1	1	0	1	0	0	0	1	0	1	1	5
Manca et al.^43^	1	1	1	1	0	0	1	1	1	1	1	8
Hosseini et al.^44^	1	1	0	1	0	0	0	1	0	1	1	5
Callesen et al.^45^	1	1	1	1	0	0	1	0	1	1	1	7
Andreu‐Caravaca et al.^46^	1	1	0	1	0	0	1	1	1	1	1	7
Ghahfarrokhi et al.^47^	1	1	1	1	0	0	1	1	1	1	1	8

Abbreviation: PEDro, Physiotherapy Evidence Database.

*Note*: 1, criterion met; 0, criterion not met; item 1 not included in total score.

^a^ Abbreviated PEDro scale item descriptions; 1, eligibility criteria specified; 2, random allocation; 3, concealed allocation; 4, groups similar at baseline; 5, participantsblinded; 6, therapists blinded; 7, assessors blinded; 8, key outcome obtained for 85% of initially allocated participants; 9, intention to treat analysis used; 10, between‐group results reported; 11, point measures and measures of variability provided.

### Effect of resistance training on walking speed

Of the 12 studies included in this review, eight reported a significant within‐group improvement for the effect of RT on at least one measure of walking speed.^37^, ^40^, ^41^, ^42^, ^43^, ^45^, ^46^, ^47^ Across these eight studies, only three included follow‐up assessments^37^, ^41^, ^43^—of which the studies by Dalgas et al.^37^ and Kjolhede et al.^41^ found that improvements in walking speed were maintained at 12 and 24 weeks postintervention, respectively. The effects of RT were compared with no‐intervention controls in six of the included studies.,^39^, ^41^, ^42^, ^44^, ^45^, ^46^ but only Kjolhede et al.^41^ found a significant between‐group difference post‐intervention in favor of the RT group. Additionally, no study found significant between changes in favor of RT when compared with active control groups; indeed one study reported a larger change in walking speed (measured using the 6MWT) in a group receiving neurofunctional training compared to RT.^47^

**FIGURE 2:**
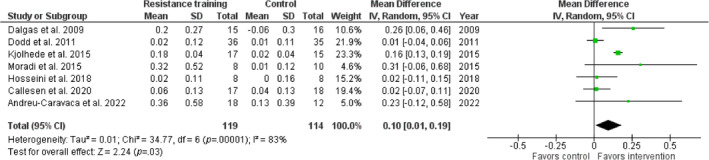
Meta‐analysis of the effect of resistance training on walking speed (m/s) across all walking outcome measures (long and short walking tests) combined. Abbreviations: CI, confidence interval; IV, initialization vector.

To allow further analysis, seven studies (including 233 participants) that reported sufficient data comparing the effects of RT with no‐intervention controls were included in a meta‐analysis.^37^, ^39^, ^41^, ^42^, ^44^, ^45^, ^46^ When the results were pooled within a random effects model, a significant improvement in walking speed of 0.10 m/s was found in favor of the intervention (*p* < .05) Figure 2. However, the intervention effect was variable (95% CI 0.01–0.19) highlighting high variability in results reported across studies. A sensitivity analysis including only results from short walking tests (10MWT, T25FWT, 50MWT)^37^, ^41^, ^42^, ^44^, ^45^, ^46^ demonstrated a larger overall effect on walking speed (0.13 m/s, 95% CI 0.04–0.23, *p* < .05). Results from long walking tests (2MWT, 6MWT)^37^, ^39^, ^41^, ^45^, ^46^ revealed a relatively smaller—although, still significant—improvement in walking speed (0.09 m/s, 95% CI 0.01–0.17, *p* < .05).

## DISCUSSION

Overall, 12 studies were included in this review, and pooled results indicate that RT significantly improves walking speed in comparison to no‐intervention controls (0.10 m/s, 95% CI 0.01–0.19). Importantly, this improvement meets the minimal important difference threshold for walking speed in MS (>0.08–0.10 m/s),^27^, ^48^ indicating a clinically significant effect. Therefore, RT should be considered as a potential treatment option for those with walking limitations as a result of MS. However, despite this positive effect, variability in results were noted across studies. Additionally, these results are mainly limited to individuals with lower levels of disability. Accordingly, further research is required to explore factors that influence the impact of RT on walking speed across the MS disease course.

One possible factor to consider in relation to the variability of intervention effect was the dose of RT prescribed. Although 11 studies^36^, ^37^, ^38^, ^39^, ^40^, ^41^, ^42^, ^44^, ^45^, ^46^, ^47^ prescribed RT in line with the currently recommend dose for people with MS (RT twice per week, performing 10–15 repetitions of each exercise over two sets),^49^ there was variation in exercise parameters resulting in different overall volumes of RT performed by participants between studies. For instance, the duration of RT interventions ranged from 6 to 24 weeks with studies prescribing between 2 and 14 sessions of RT per week. Furthermore, the volume (sets/repetitions) and intensity of RT within each session differed, with seven studies varying volume throughout the intervention in accordance with progression of intensity.^37^, ^38^, ^41^, ^42^, ^45^, ^46^, ^47^ Although dose response cannot be inferred from the results of this review, a previous systematic review suggests progressively increasing RT intensity from 50% to 90% of 1 repetition maximum significantly improves muscle function after 24 weeks.^50^ This supports earlier recommendations for RT prescription in MS that also highlight progressively increasing the number of sets per exercise from 1–3 to 3–4.^51^ Considering the importance of lower limb muscle function to walking capacity,^22^ prescribing RT at a sufficient dose to improve muscle function may also positively affect walking speed. Therefore, future research should determine the optimal prescription of RT to enhance walking speed and evaluate, for instance, whether higher doses of RT have a greater effect on walking outcomes alongside muscle function.^52^

In addition to different doses of RT being prescribed across studies, there was also variability in the mode of RT and muscle groups targeted. The American College of Sports Medicine recommends that RT should be specific to the muscle groups being trained alongside the muscle action and range/speed of movement.^53^ In relation to RT for walking speed in people with neurological conditions, it is recommended that training specifically targets the muscles with a key responsibility for forward propulsion during walking^54^—namely the ankle plantar flexors, hip flexors, and hip extensors.^55^, ^56^, ^57^ However, the most common muscle group targeted by the studies included in this review was the knee extensors, with only seven studies including RT exercises for ankle plantar flexors.^39^, ^40^, ^41^, ^44^, ^45^, ^46^, ^47^ Furthermore, only two of the studies that included plantarflexion exercises reported performing these in a closed‐chain position.^39^, ^45^ Although knee extensor strength is associated with overall walking capacity,^22^ it has low‐moderate correlation with walking speed—particularly in those with lower levels of walking limitations (such as the participants included in this review).^58^, ^59^ Therefore, to improve walking speed in people with MS, future studies should consider the mode of RT alongside the muscle groups included to ensure specificity of training to the target outcome.

Whereas previous systematic reviews have investigated the effect of RT on overall walking capacity in MS,^23^, ^24^, ^25^, ^26^ this is the first meta‐analysis to report results specific to walking speed. As highlighted by the evidence included within this review, walking speed can be measured using various methods. For instance, six studies included in this review measured walking speed using short walking tests (10MWT, T25FWT, 50MWT),^37^, ^41^, ^42^, ^44^, ^45^, ^46^ whereas five others used long walking tests (2MWT, 6MWT).^37^, ^39^, ^41^, ^45^, ^46^ The use of short tests for estimating walking speed in MS is limited particularly in those with mild disability and walking limitations due to lower sensitivity compared to longer walking tests.^60^, ^61^ Alternatively, although longer walking tests may be more sensitive to change, these tests are primarily intended to measure walking endurance and variability in walking speed across the duration of the test and may result in a less accurate representation of walking speed.^62^ Sensitivity analysis of the results included within this meta‐analysis showed that whereas significant improvements in walking speed were found using both short and long tests, a larger effect was reported across shorter tests. However, alongside the type of walking test used to measure walking speed, future studies should also consider how variability in walking speed—over both long and short distances—translate to an individual's usual environment and activities of daily living.^63^

Importantly, the results of this meta‐analysis focused on the effects of RT alone in comparison to no‐intervention controls. Therefore, the effectiveness of RT compared to other forms of exercise or rehabilitation cannot be concluded from this review. Although seven studies included in this review compared the effects of RT with other interventions, only one found that another form of rehabilitation/exercise—neurofunctional training—had a significantly greater effect on walking speed compared to RT.^47^ Due to the multifactorial nature of walking limitations,^64^ future studies should consider whether complex interventions—combining RT with other forms of rehabilitation—may have a greater effect on walking limitations such as walking speed, compared to RT alone.

### Limitations

There are several limitations to consider when interpreting the findings of this review. Small sample sizes and heterogenous results reported across studies limit the precise estimation of the possible effect of RT on walking speed in people with MS. Additionally, most studies recruited people with RR MS and/or participants with mild–moderate disability; this limits the generalizability of the results to those with progressive forms of MS and/or those with higher levels of disability and introduces a possible floor effect due to minimal walking limitations at baseline. Future research should investigate whether similar effects of RT are noted in people with higher levels of disability and walking limitations. Lastly, due to the aims of this review, several studies that evaluated the effect of RT on other aspects of walking in MS (eg, overall functional mobility, subjective self‐reported walking measures) were excluded from this review. Therefore, this review reports the potential effect of RT on walking speed alone—not on overall walking ability.

## CONCLUSIONS

This systematic review and meta‐analysis demonstrates that RT significantly improves walking speed in comparison to no‐intervention controls (0.10 m/s, 95% CI 0.01–0.19) in people with MS. However, variability in the results were noted—particularly when compared to other forms of exercise. Therefore, future research should investigate the optimum type and dose of RT alongside the transferability of changes in walking speed to an individual's usual activities and environment.

## FUNDING INFORMATION

This research received no specific grant from any funding agency in the public, commercial, or not‐for‐profit sectors.

## DISCLOSURE

All authors report that they have no conflicts of interest.

## Supporting information


**Data S1.** Supporting Information.
