# Supplementary material for: Changes in walking speed following resistance training in people with multiple sclerosis: A systematic review and meta‐analysis
Source: PM R. 2024 Sep 23;17(2):222–37. doi: 10.1002/pmrj.13255 (PMC11826970; doi:10.1002/pmrj.13255)
Supplement: Supplementary file 1 — Data S1. Supporting Information. [file PMRJ-17-222-s001.docx]

**Supplementary material 1: Literature Search Strategies**

**CINAHL**

1. (MH “Multiple Sclerosis”) OR “multiple sclerosis”
2. (MH "Resistance Training") OR "resistance training" OR (MH "Muscle Strengthening")
3. “resistance exercise*”
4. “strength training”
5. “strength exercise*”
6. “strength program”
7. 2 OR 3 OR 4 OR 5 OR 6
8. (MH "Walking") OR "walking" OR (MH "Walking Speed")
9. “walking ability”
10. “walking performance”
11. “walking capacity”
12. (MH “Gait”) OR “gait”
13. “mobility”
14. “ambulation”
15. “physical function”
16. “functional ability”
17. “functional capacity”
18. 8 OR 9 OR 10 OR 11 OR 12 OR 13 OR 14 OR 15 OR 16 OR 17
19. 1 AND 7 AND 18

**MEDLINE**

1. (MH “Multiple Sclerosis”) OR “multiple sclerosis”
2. (MH "Resistance Training") OR "resistance training"
3. “muscle strengthening”
4. “resistance exercise*”
5. “strength training”
6. “strength exercise*”
7. “strength program”
8. 2 OR 3 OR 4 OR 5 OR 6 OR 7
9. (MH "Walking") OR "walking" OR (MH "Walking Speed")
10. “walking ability”
11. “walking performance”
12. “walking capacity”
13. (MH “Gait”) OR “gait”
14. “mobility”
15. “ambulation”
16. “physical function”
17. “functional ability”
18. “functional capacity”
19. 9 OR 10 OR 11 OR 12 OR 13 OR 14 OR 15 OR 16 OR 17 Or 18
20. 1 AND 8 AND 19

**AMED (The Allied and Complimentary Medicine Database)**

1. multiple sclerosis
2. resistance training
3. muscle strengthening
4. resistance exercise*
5. strength training
6. strength exercise*
7. strength program
8. 2 OR 3 OR 4 OR 5 OR 6 OR 7
9. Walking
10. Walking speed
11. Walking ability
12. Walking performance
13. Walking capacity
14. Gait
15. Mobility
16. Ambulation
17. physical function
18. functional ability
19. functional capacity
20. 9 OR 10 OR 11 OR 12 OR 13 OR 15 OR 16 OR 17 OR 18 OR 19
21. 1 AND 8 AND 20

**Web of Science:**

1. “Multiple sclerosis”
2. “resistance training” OR “muscle strengthening” OR “resistance exercise*” OR “strength training” OR “strength exercise*” OR “strength program”
3. “walking” OR “walking speed” OR “walking ability” OR “walking performance” OR “walking capacity” OR “gait” OR “mobility” OR “ambulation” OR “physical function” OR “functional ability” OR “functional capacity”
4. 1 AND 2 AND 3

**PEDro (Physiotherapy Evidence Database):**

1. “Multiple Sclerosis” AND “resistance*” AND function*

**PsycINFO:**

1. (Multiple sclerosis) AND (resistance training OR muscle strengthening OR resistance exercise* OR strength training OR strength exercise* OR strength program) AND (walking OR walking speed OR walking ability OR walking performance OR walking capacity OR gait OR mobility OR ambulation OR physical function OR functional ability OR functional capacity)

**Sports Medicine & Education Index:**

Filters Used: in Abstract

1. (Multiple sclerosis) AND (resistance training OR muscle strengthening OR resistance exercise* OR strength training OR strength exercise* OR strength program) AND (walking OR walking speed OR walking ability OR walking performance OR walking capacity OR gait OR mobility OR ambulation OR physical function OR functional ability OR functional capacity)
